# Anti-CGRP in cluster headache therapy: a response

**DOI:** 10.1007/s10072-020-04335-3

**Published:** 2020-04-08

**Authors:** Richard Wenzel, Jennifer Bardos, Sheena Aurora

**Affiliations:** 1 Migraine and Headache Disorders, Lilly Bio-Medicines, Indianapolis, IN USA; 2 Clinical Pain, Lilly Bio-Medicines, Indianapolis, IN USA; 3 Migraine and Pain Team, Lilly Bio-Medicines, Indianapolis, IN USA

Dear Editor in Chief,

Your recently published article, *Anti-CGRP in cluster headache therapy* (May 2019;40(suppl 1):S129-S135), helps foster greater awareness about, and treatment of, cluster headache (CH). These important goals are best met via accurate, up-to-date information, thus please consider the following:

1. On page S132 the article states, “*Two trials exploring anti*-*CGRP monoclonal antibodies efficacy for the prevention of chronic CH*, *galcanezumab NCT02438826 and fremanezumab NCT02964338*, *stopped recruitment because futility analysis revealed that the primary endpoint was unlikely to be met.*” In actuality, this galcanezumab chronic CH study attained the protocol’s intended enrollment population, thus is a completed trial. This study’s primary endpoint was not met. Results for the double-blind treatment phase are currently available via Clinicaltrials.gov or an abstract [1], and a manuscript describing these results has been submitted to a peer-reviewed journal.
2. Also on page S132, regarding the phase III galcanezumab episodic CH trial (NCT02397473), the article states, “*The proportion of CH patients showing* ≥ *50*% *reduction in weekly CH attack frequency at week 3 was 76*% *for galcanezumab compared to 57*% *for placebo* (*p = 0.04*)*.*” At the time of your article’s publication, this information was accurate, but please be aware that the trial’s sponsors subsequently updated this data, which is now available in the published primary manuscript [2], “*The key secondary end point of the percentage of patients having a reduction of at least 50*% *in the weekly frequency of cluster headache attacks at week 3 was 71*% *in the galcanezumab group*, *as compared with 53*% *in the placebo group* (*p = 0.046*).”

Thank you for your consideration.

On behalf of Eli Lilly & Company,
